# Polycystic Ovary Syndrome: Past, Present and Future

**DOI:** 10.3390/jcm12113808

**Published:** 2023-06-01

**Authors:** Blazej Meczekalski

**Affiliations:** Department of Gynecological Endocrinology, Poznan University of Medical Sciences, Polna 33, 60-535 Poznan, Poland; blazejmeczekalski@ump.edu.pl

Early mentions of PCOS as a disorder can be traced back to ancient history. In his groundbreaking work “Diseases of Women”, Hippocrates (460 BC-377 BC) noted, “But those women whose menstruation is less than three days or is meagre, are robust, with a healthy complexion and a masculine appearance; yet they are not concerned about bearing children nor do they become pregnant” [1].

Now, what about modern medicine and PCOS?

Modern science has studied PCOS for the greater part of the last 150 years [2]. Initial observations of the ovarian appearance and clinical symptoms characteristic of the disorder have been studied long before Stein and Levental published their observations [3] in 1935. For instance, Dr. Achille Chereau in his 1844 book on the diseases of the ovaries wrote, “Ovaries are enlarged in volume, elastic, with smooth and shiny surface containing many small cysts, located on the periphery of the organ” [4]. There are many examples in the historical literature prior to 1935 of researchers from both Europe and the United States who described ovaries by focusing on their gross pathological characteristics [2].

From 1935 to the end of the 1970s, the research work was focused mainly on the endocrine background of PCOS. The last two decades of the 20th century saw this focus shift to metabolic problems associated with PCOS [5], while in recent years we have seen advanced techniques in molecular biology derive novel insight into its underlying pathophysiology.

One challenge currently facing this condition is its misleading name. Historically, “Polycystic ovary syndrome” was first coined by Stein and Leventhal after their gross observation of ovary specimens. Other names include “Polycystic ovarian syndrome”, “Polycystic ovary disease”, and, in earlier publications, “Stein-Leventhal Syndrome”. These conventional names are misleading, confusing, and generally project a negative image onto patients [5]. It is now understood that patients with PCOS whose ovaries have such a morphology do not in fact have cysts on the ovaries, but rather follicles arrested in the early stages of development as a result of hyperandrogenemia. Conceivably, renaming the syndrome would be easier than uncovering its true etiopathogenesis. However, as we continue to strive ever closer to unlocking its underlying mechanism, expending efforts at the present time on renaming it with only a placeholder would be inappropriate. It would be much more productive to channel efforts into further understanding the background of PCOS (in both reproductive and metabolic problems) to enable the development of better, more reliable diagnostic criteria and treatments.

As was previously mentioned, we have now entered the era of molecular research of studying PCOS. Despite ongoing decades of research, it has remained a challenge to elucidate the cellular and molecular background of PCOS. Zhanh et al. [6] suggested that the heterogeneity of PCOS manifestations is reflected in different mechanistic pathways, and these will finally be identified using a genetic approach. Bizon et al. [7] assessed for the first time the rs2070424 polymorphism of superoxide dismutase (SOD1) in women with PCOS, detecting a dominant variant, AA (93.3%). 

One big outstanding piece of the PCOS pathophysiology puzzle is the metabolic question. Although metabolic parameters are not explicitly included in the current Rotterdam Criteria of PCOS, studies on the metabolic influence and implications have brought to light its critical importance [8,9]. 

Lu et al. [10] reported that serum RANKL correlates positively with an increased risk of nonalcoholic fatty liver disease (NAFLD) in Chinese women with PCOS. This observation was found to be independent of metabolic and reproductive factors. We cannot ignore the significance and role of neuroendocrine pathophysiology in PCOS and the potential for the use of treatments which interfere with the neuroendocrine profile [11]. Promising results have emerged in the use of new drugs—NK3R antagonists, or kappa receptor agonists. This new class of drug has been shown to decrease the activity in GnRH pulse generation (PCOS is characterized by an increase in GnRH pulse frequency) and, in turn, can decrease serum testosterone levels by up to 30% [12].

Beyond the enigma of the pathophysiology of PCOS, we should look to solve important problems of secondary order in the future. These problems include: clearly outlining the presentation of PCOS in adolescents, establishing how PCOS affects and course in pregnancy, and defining the effect of PCOS on menopause and in postmenopausal women.

The question of PCOS in adolescence is of particular interest. Very few studies have been undertaken to address the shortcomings in the diagnosis and treatment models involving adolescents. Randomized double-blind placebo-controlled studies evaluating the use of metformin and combined oral contraceptives in adolescent PCOS patients are virtually nonexistent. We are therefore left to independently consider the safety of using combined oral contraceptives in these patients in relation to neuroendocrine system function, bone health status (peak bone mass acquisition), and its effect on the cardiovascular system. The choice of optimal pharmacotherapy in addressing PCOS in adolescents often involves much ambiguity and is subject to considerable controversy [13].

How do we manage pregnant women who have a history of PCOS?

Recent studies have shown that such pregnant women have a significantly increased risk of miscarriage, gestational diabetes, preeclampsia, and pregnancy-induced high blood pressure. Therefore, it is very important to initiate pre-pregnancy screening and monitoring for overweight and obese women with PCOS [14]. Awareness regarding obstetric challenges should increase, whereas further research should be undertaken to better understand this group of patients. Currently, we have no guidelines on antenatal care which specifically address women with PCOS. Most current recommendations are indirect and nonspecific. Updated statements and clinical guidelines are desperately needed and should be prepared by scientific societies on the topic of gestational care (addressing both maternal and fetal aspects) in the context of PCOS as a high-risk pregnancy. 

The broad and intertwining relationships between PCOS, aging, and menopause require clarification. Jacewicz-Swiecka et al. [15] observed that progressing from the third to the fourth decade of life is often associated with a reduction in PCOS features, and also appears to have a great impact on the overall fertility of women with a previous established diagnosis of PCOS. 

The question remains to what extent does PCOS alone increase the risk of cardiovascular disease in postmenopausal women. We know that although aging ameliorates the clinical manifestation of PCOS (hyperandrogenization and metabolic abnormalities), PCOS persists beyond menopause. On the other side, menopause increases the risk of CVD in the general population [16]. Large prospective studies on community-based and well-phenotyped PCOS cohorts with extended follow-up into late menopause are needed to explore these outcomes.

From a broader perspective, PCOS is predominantly perceived by patients and clinicians as an issue affecting fertility in women of reproductive age. This represents a critical concern, and numerous studies are currently being conducted in this area. For instance, a recent study by Holzer et al. [17] reported on their success in using letrozole for ovulation induction in women with PCOS, determining it to be independent from traditional calcium-associated signaling pathways and other parameters of calcium metabolism. 

However, our focus on PCOS should not be limited solely to issues such as menstrual disorders, hyperandrogenization, and infertility in women of reproductive age. It is crucial to bear in mind that PCOS encompasses a distinct multidimensional aspect, which extends to adolescent patients, pregnant individuals, and postmenopausal women as well.
